# Targeting the Fibroblast Growth Factor Receptor (FGFR) in Advanced Cholangiocarcinoma: Clinical Trial Progress and Future Considerations

**DOI:** 10.3390/cancers13071706

**Published:** 2021-04-03

**Authors:** Patrick C. Lee, Andrew Hendifar, Arsen Osipov, May Cho, Daneng Li, Jun Gong

**Affiliations:** 1 Department of Medicine, Cedars-Sinai Medical Center, Los Angeles, CA 90048, USA; Patrick.Lee2@cshs.org (P.C.L.); Andrew.Hendifar@cshs.org (A.H.); Arsen.Osipov@cshs.org (A.O.); 2 Department of Medicine, UC Davis Comprehensive Cancer Center, Sacramento, CA 95817, USA; mayc5@hs.uci.edu; 3 UCI Health Chao Family Comprehensive Cancer Center, University of California Irvine, Orange, CA 92868, USA; 4 Department of Medical Oncology, City of Hope Comprehensive Cancer Center, Duarte, CA 91010, USA; danli@coh.org

**Keywords:** cholangiocarcinoma, fibroblast growth factor receptor (FGFR), pemigatinib, infigratinib, derazantinib, debio 1347, futibatinib, TAS-120, erdafitinib

## Abstract

**Simple Summary:**

Cholangiocarcinoma (CCA) is a cancer arising from the bile ducts. Chemotherapy has long been the standard of care for metastatic CCA, but recent clinical trials have shown that fibroblast growth factor receptor (FGFR) inhibitors are a promising new treatment in advanced CCA with documented genetic alterations in *FGFR* genes. This review provides an overview of the genetic features of CCA, the biology of the FGFR pathway, important FGFR inhibitor clinical trials in CCA, and future opportunities and challenges in the development of FGFR inhibitors for effective clinical use in patients with CCA.

**Abstract:**

Landmark molecular profiling efforts have identified multiple targetable alterations in cholangiocarcinoma. Among the molecular-driven subsets of cholangiocarcinoma, targeting the fibroblast growth factor receptor (FGFR) has shown promise and represents the first targeted therapy to be approved in treatment-refractory, advanced cholangiocarcinoma. In this review, we provide an up-to-date overview of the clinical development of FGFR inhibitors in advanced cholangiocarcinoma. We review the FGFR pathway and discuss emerging issues including resistance to FGFR inhibitors. We end with a discussion on future considerations to optimize the potential of this class of therapeutics in advanced cholangiocarcinoma.

## 1. Introduction

Cholangiocarcinomas (CCAs) are comprised of a heterogenous group of cancers that arise from the intra- or extrahepatic bile ducts and constitute the second most common primary liver tumor behind hepatocellular carcinoma [1,2]. Although globally, CCA represents a rare cancer accounting for approximately 3% of all gastrointestinal cancers and an incidence of <6 cases per 100,000 people, there is an exquisite high incidence rate in certain countries including Chile, Bolivia, South Korea, and North Thailand [1,3]. CCA is classically categorized into intrahepatic (iCCA) and extrahepatic cholangiocarcinoma (eCCA) with separation by the second order bile ducts [1]. Extrahepatic CCA can be further divided into hilar (Klatskin) or perihilar and distal tumors, as separated by the insertion of the cystic duct. Specifically, perihilar CCA arises in the right and/or left hepatic duct or at their junction, distal CCA involves the common bile duct, and iCCA arises above the second-order bile ducts. The majority of CCAs are hilar (~60%) followed by distal tumors (20–30%) and intrahepatic tumors (5–10%) although there has been a progressive global increase in incidence and mortality for iCCA [1,2,4,5]

Less than one-third of cases of CCA present with resectable disease, in which surgery represents the only potential curative intent treatment for CCA [6]. Five-year overall survival (OS) rates following surgical resection range from 22 to 44% for iCCA, 11 to 41% for perihilar CCA, and 27 to 37% for distal CCA [6]. Unfortunately, for the majority of cases that present with locally advanced, unresectable or metastatic disease, treatment is palliative and comprised of systemic therapy with a median OS of 11.7 months when treated with cisplatin and gemcitabine per the phase III ABC-02 trial, the recognized standard regimen in the first-line setting [7]. Beyond the first-line setting for advanced CCA, there is no established second-line therapy except for preliminary data from the phase III ABC-06 trial supporting the use of 5-fluorouracil and oxaliplatin (FOLFOX) in those previously treated with cisplatin and gemcitabine [8]. However, landmark comprehensive molecular profiling efforts in CCA have identified a subset of CCAs with targetable alterations in the fibroblast growth factor receptor (*FGFR*), which have resulted in the development of FGFR-directed therapies as an established and viable class of therapeutics in the treatment paradigm of refractory CCA. In this review, we will highlight the molecular landscape of *FGFR* alterations in CCA. We detail the clinical development of FGFR inhibitors in advanced CCA. We end with a discussion on future considerations for this promising class of targeted therapies in advanced CCA.

## 2. Molecular Landscape of Cholangiocarcinoma

Prior to the U.S. Food and Drug Administration (FDA) approval of the FGFR inhibitor pemigatinib in April 2020 in treatment-refractory CCA, there was no approved targeted therapy in CCA. However, it has been increasingly recognized that CCA represents a molecularly driven tumor type with recent large-scale comprehensive genomic profiling having identified a multitude of targetable alterations and prognostic subgroups [8,9]. The most common genetic alterations in CCA are *IDH1* (30%), *ARID1A* (23%), *BAP1* (20%), *TP53* (20%), and *FGFR2* gene fusions (14%) with less frequent mutations in *PIK3CA*, *NRAS*, and *ERBB2*, as assessed within a cohort of 195 patients by targeted sequencing of 410 cancer-associated genes [10]. In particular, *IDH1* and *FGFR2* were deemed to be the two primary actionable genes (OncoKB classification level 3 or higher). Identification of such alterations is paving the way for better stratification and prognostication of CCA. Within a cohort of 496 iCCA tumors, stratification by mutational status of *KRAS*, *TP53*, and *IDH* distinguished unique oncogenic programs and sensitivities to high-throughput drug panels [11]. In another study, whole-exome sequencing was performed on 239 biliary tract cancers (137 iCCA, 74 eCCA, and 28 gallbladder cancers) [12]. The authors identified subtype-specific alterations in different growth factor-mediated signaling pathways, most notably *FGFR1/2* alterations, which occurred only in iCCA, and protein kinase A (*PKA*) alterations, which occurred in both iCCA and eCCA. A subset of 160 tumors also underwent transcriptome sequencing and were categorized via unsupervised clustering into four subgroups, which happened to correlate with patient prognosis. The best prognosis group was enriched for eCCA and had negative enrichment of RAS and MAPKK activation signatures, while the worst prognosis group was associated with high mutational burden and increased antiapoptotic, cytokine, and immune gene signatures, including higher expression of immune checkpoint molecules.

Integration of multi-omic data has provided additional insight into CCA subtypes. In a proof-of-principle analysis of 38 fluke-negative CCA samples from The Cancer Genome Atlas (TCGA), unsupervised hierarchical clustering of mRNA expression data identified a subtype enriched for *IDH*-mutant samples, which demonstrated increased mitochondrial gene expression and decreased chromatin modifier gene expression [9]. Cross-correlation with a methylation dataset implicated hypermethylation and corresponding downregulation of the chromatin modifier *ARID1A* in particular. Moreover, an integrative whole-genome, exome, copy number, expression, and methylation analysis of 489 CCAs (133 fluke-positive, 356 fluke-negative) revealed 4 unique CCA clusters. Clusters 1 and 2 were mostly fluke-positive tumors, characterized by *TP53* mutations, *ERBB2* amplifications, and CpG island hypermethylation, while clusters 3 and 4 were primarily fluke-negative tumors that exhibited enrichment in expression of immune pathway-related genes including *PD1* and *PDL2* (cluster 3) or in *IDH1/2* mutations, *BAP1* mutations, *FGFR* alterations, and CpG shore hypermethylation (cluster 4) [13]. This molecular heterogeneity highlights a need for further clinical stratification beyond anatomical location.

Molecular profiling efforts by multiple groups have ultimately corroborated that CCA, particularly iCCA, harbor alterations in *IDH1/2*, *FGFR2*, *ERBB2*, *EGFR*, *BRAF*, *MET*, *BRCA1/2*, and *NTRK* with viable classes of agents available for therapeutic targeting [14,15,16,17,18,19,20]. One such target, IDH1 (detected in approximately 13% of iCCAs), has recently been therapeutically targeted in the randomized, phase III ClarIDHy trial [21]. Here, 185 patients with metastatic *IDH1*-mutant CCA refractory to up to two previous lines of gemcitabine-based or fluorouracil-based chemotherapy were randomized to receive the oral IDH1 inhibitor ivosidenib or placebo. A superior progression-free survival (PFS) benefit was seen with ivosidenib (median 2.7 months) over placebo (1.4 months, hazard ratio (HR) 0.37, 95% confidence interval (CI) 0.25–0.54, one-sided *p* > 0.0001). With regard to notable secondary endpoints, there was not a statistically significant difference in median overall survival, and the objective response rate was 2% in the ivosidenib group compared to 0% in placebo. Although ivosidenib is seeking FDA approval in Q1 2021 based on the results of ClarIDHy, the first class of targeted drugs approved in treatment-refractory, advanced CCA were FGFR inhibitors. We will focus on an overview of the FGFR inhibitors that have undergone the most investigation thus far in advanced CCA with a discussion on integration of FGFR inhibitors into clinical practice.

## 3. Fibroblast Growth Factor Receptor

**Figure 1:**
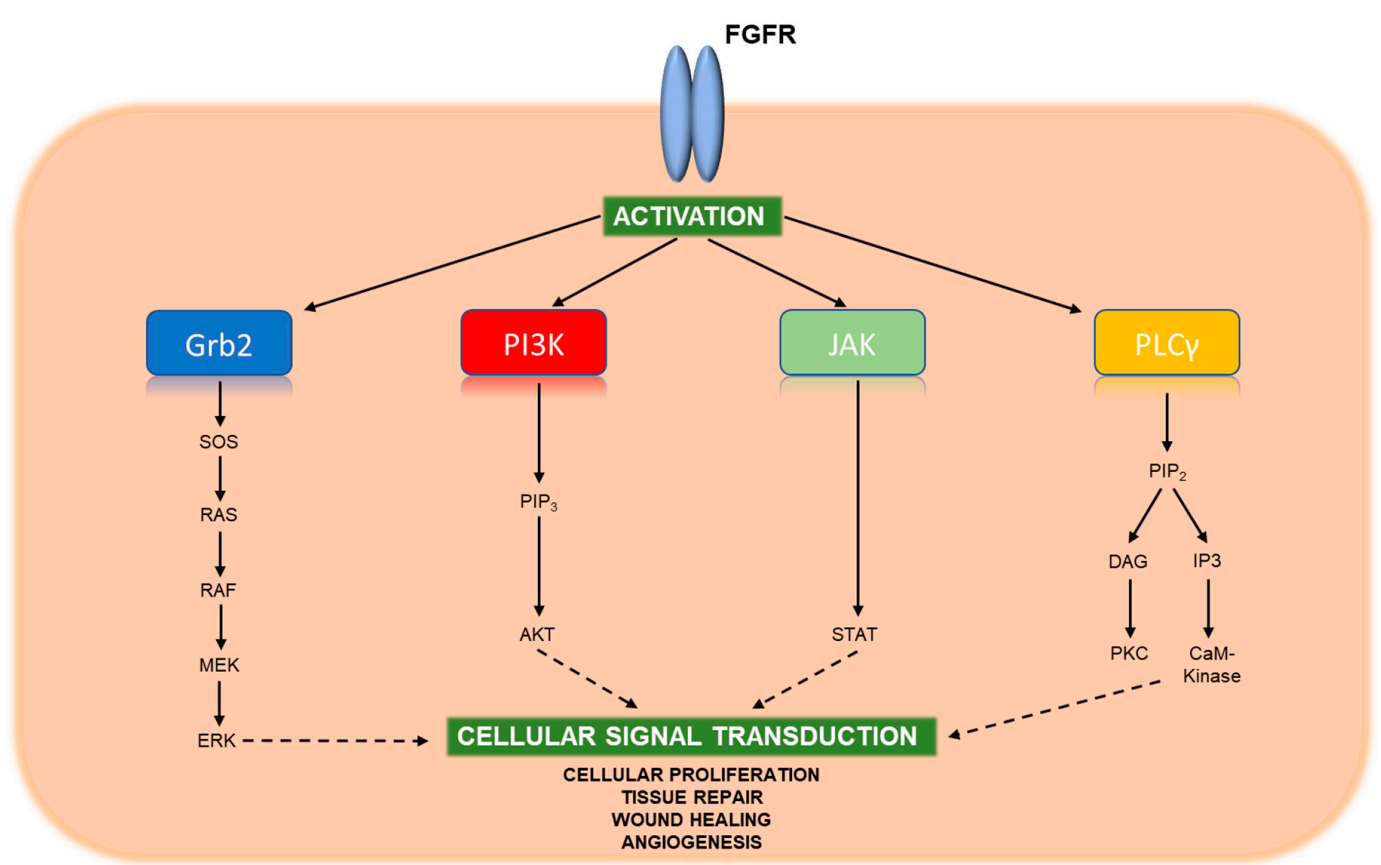
Key signaling pathways of activated FGFR. Upon binding FGF ligands, FGFR molecules dimerize and undergo cross-phosphorylation of tyrosine residues within their activation loops located near the cytoplasmic tail. This phosphorylation activates the kinase domain, which in turn binds and phosphorylates adaptor proteins of downstream signaling pathways. Four particularly important pathways include RAS-MAPK, PI3K-AKT, JAK-STAT, and PLCγ, which contribute to cellular proliferation, tissue repair, wound healing, and angiogenesis.

The fibroblast growth factor receptors (FGFRs), comprising FGFR1-4, are a family of receptor tyrosine kinases (RTKs) that play important roles in embryonic development, tissue repair, and tumor angiogenesis and proliferation [22]. The extracellular domain of FGFRs can bind 22 different fibroblast growth factors (FGFs) depending on the cellular context [23]. The binding of FGF to FGFR induces conformational changes leading to FGFR dimerization and mutual cross-phosphorylation of tyrosine residues on their cytoplasmic tails. This activates intracellular kinase domains, which subsequently phosphorylate adaptor proteins leading to activation of several downstream signaling pathways including Ras-Raf-MAPK, PI3K-AKT, JAK-STAT, and PLCγ (Figure 1) [24]. While *FGFR1-4* alterations have been described in many cancers, *FGFR2* fusions are particularly enriched in iCCA, occurring in 5–15% of cases, although there have been reports as high as 45% [17,19,25,26,27,28,29]. *BICC1* is by far the most common fusion partner, and other recurrent partners include *PPHLN1*, *AHCYL1*, and *CCDC6* [19,30,31,32]. Overall, some of the larger iCCA datasets have detected up to 63 unique fusion partners within a cohort [31]. Structurally, these fusions often combine the N-terminus of FGFR2, including an intact intracellular tyrosine kinase domain, with the C-terminus of a protein that contains an oligomerization domain. This oligomerization domain mediates ligand-independent dimerization of FGFR2 fusion proteins and results in constitutive downstream activation [28,33].

## 4. Clinical Development of FGFR Inhibitors in Advanced Cholangiocarcinoma

A host of small-molecule FGFR inhibitors are currently in various stages of clinical development (Table 1). Phase I and II studies have generally demonstrated excellent safety profiles and promising results with regard to efficacy and therapeutic potential. Additionally, several phase III studies are already underway. Clinical trial results for several promising FGFR inhibitors are summarized below.

### 4.1. Pemigatinib

The selective FGFR1-3 inhibitor pemigatinib became the first FDA-approved FGFR inhibitor for treatment-refractory, advanced CCA harboring *FGFR2* fusions or rearrangements based on the FIGHT-202 trial [34]. This was a phase 2, single-arm trial of 146 CCA patients with locally advanced or metastatic disease who had failed at least one prior systemic therapy. Patients were scheduled to receive 13.5 mg oral pemigatinib once daily on a 21-day cycle (two weeks on, one week off). The study included 107 patients with *FGFR2* fusions or rearrangements, 20 with other *FGF/FGFR* alterations, 18 with no *FGF/FGFR* alterations, and one with an undetermined *FGF/FGFR* alteration. The primary endpoint was overall response rate (ORR) within the *FGFR2* fusion/rearrangement subgroup, which was 35.5% (95% CI 26.5–45.4%), including 3 CRs and 35 PRs, at a median follow-up of 17.8 months. None of the patients with other *FGF/FGFR* alterations or without *FGF/FGFR* alterations achieved an objective response. A notable secondary endpoint was progression-free survival, which was 6.9 months (95% CI 6.2–9.6) within the *FGFR2* fusion/rearrangement group. The most common adverse event (AE) was hyperphosphatemia (60% of patients), which is an on-target side effect of FGFR inhibitors. Other less frequent (<=12%) AEs include hypophosphatemia, arthralgia, stomatitis, hyponatremia, abdominal pain, and fatigue. 64% of patients experienced grade 3 or worse AEs, most commonly hypophosphatemia in 12% of patients, while all hyperphosphatemia events were grade 1 or 2.

### 4.2. Infigratinib

Infigratinib (BGJ398) is another pan-FGFR, ATP-competitive inhibitor. In a Phase II study conducted in patients with advanced or metastatic CCA with *FGFR2* fusions or rearrangements who had failed at least one prior systemic therapy, patients received infigratinib 125 mg once daily in 28-day cycles (3 weeks on, 1 week off) [35,36,37]. A cohort analysis of 108 patients was reported on 31 March 2020 [35]. With regard to primary endpoints, ORR was 23.1% (95% CI 15.6–32.2) with a median duration of response (mDOR) of 5.0 months (range 0.9–19.1). The median PFS was 7.3 months (95% CI 5.6–7.6). A prespecified subgroup analysis showed that patients who had received second-line infigratinib achieved an even higher ORR of 34% [35]. Hyperphosphatemia was the most common AE (76.9%), for which patients received prophylaxis with the phosphate binder sevelamer. Others included eye disorders (67.6%), stomatitis (54.6%), and fatigue (39.8%). Common grade 3/4 adverse events were stomatitis (14.8%), hyponatremia (13.0%), hypophosphatemia (13.0%), and hyperphosphatemia (10.3%, all grade 3). Central serous retinopathy/retinal pigment epithelium detachment (CSR/RPED) did occur in 16.7% of patients, one case of which was grade 3.

### 4.3. Derazantinib

Another pan-FGFR inhibitor, derazantinib, was tested in a Phase I/II, open-label study (ARQ 087-101) in 29 patients with unresectable, *FGFR2* gene fusion-positive iCCA, including 27 who progressed after at least one systemic therapy and 2 treatment-naïve who were ineligible for standard first-line therapy [38]. Patients received either 300 mg (n = 27) or 400 mg (n = 2) once daily continuously without interruption. While the primary endpoint was safety and tolerability, notable secondary endpoints include an ORR of 20.7% (0 CRs, 6 PRs) and disease control rate of 82.8%, with a median PFS of 5.7 months (95% CI 4.0–9.2). Common AEs included hyperphosphatemia (75.9%), asthenia/fatigue (69.0%), and eye toxicity (41.4%). Grade 3 or higher adverse events were reported in 27.6% of patients. A post hoc analysis of ARQ 087-101 also suggested efficacy of derazantinib in patients with *FGFR2* mutations and amplifications, with similar mPFS compared to *FGFR2* fusions [39], albeit in a small sample size. Based on this, the Phase II FIDES-01 trial is recruiting both *FGFR2* fusion and *FGFR2* mutation/amplification cohorts [48]. A preliminary pooled analysis of 20 patients with *FGFR2* mutations/amplifications from ARQ 087-101, FIDES-01, and early access programs demonstrated a mPFS of 8.1 months (95% CI 4.6–14.8) [49].

### 4.4. Debio 1347

A first-in-human Phase I study of the FGFR inhibitor Debio 1347 was conducted in patients with advanced solid malignancies harboring activating *FGFR* alterations, including 9 iCCA patients (5 *FGFR2* translocations and one each of the following: *FGFR1* translocation, *FGFR2* mutation, *FGFR2* activating deletion, and *FGFR3* mutation) [40,41]. Patients received escalating doses of Debio 1347 until disease progression or side effect intolerance, between 60 and 150 mg oral once daily on continuous 28-day cycles. Within the iCCA group, 2/9 (22.2%) patients had a partial response (PR), while 4/9 (44.4%) had stable disease. Within the entire cohort of 58 patients, the most frequent AEs were hyperphosphatemia (76%), diarrhea (41%), nausea (40%), fatigue (38%), and constipation (33%). Based on this toxicity profile, 80 mg was determined to be the maximally tolerated dose. 63% of patients experienced a grade 3 or higher adverse event, including 21% with grade 3 or higher hyperphosphatemia. The Phase II FUZE trial of Debio 1347 is currently ongoing.

### 4.5. Futibatinib (TAS-120)

Futibatinib is an irreversible FGFR1-4 inhibitor that notably has shown efficacy in cell lines displaying resistance to other FGFR inhibitors [25]. The phase I FOENIX-101 trial investigated futibatinib in advanced solid tumors, including 45 CCA patients (62% *FGFR2* gene fusions, 38% other *FGF/FGFR* alterations). All CCA patients had received prior systemic therapy, including 13 who had received at least one reversible FGFR inhibitor [42]. Patients were treated at either 16, 20, or 24 mg daily. ORR was 25% (7 of 28) in those with *FGFR2* gene fusions and 17.6% in those with other alterations. Notably, 4 of 13 patients who had been previously treated with other FGFR inhibitors displayed PR. The most common AEs included hyperphosphatemia (78%), increased aspartate aminotransferase (29%), dry skin (29%), diarrhea (27%), and dry mouth (27%). Grade 3 hyperphosphatemia occurred in 27% of patients, with no grade 4 or 5 treatment-related adverse events. The Phase II FOENIX-CCA2 trial, limited to iCCA patients with FGFR2 fusions or rearrangements who have received prior systemic therapy but not including prior FGFR inhibitors, is currently ongoing. A planned interim analysis of 67 patients revealed an ORR of 37.3% with median PFS of 7.2 months, similar to pemigatinib and infigratinib [43,44].

### 4.6. Erdafitinib

Erdafitinib is another potent FGFR1-4 inhibitor. The first-in-human phase I study of erdafitinib enrolled 187 patients with advanced solid tumors who had failed standard therapy [46]. The study employed a 4 part design, with part 1 involving dose escalation, while parts 2 to 4 involved molecular screening for activating *FGFR* genomic alterations. Within response-evaluable patients with *FGFR* mutations or fusions, cholangiocarcinoma exhibited the second highest ORR of all cancers (27.3%, 3 of 11), behind urothelial carcinoma (46.2%, 12 of 26). The median duration of response was 12.9 months for the three partial responders, while median progression-free survival was 5.1 months (95% CI 1.6–16.4) [45]. In the phase IIa LUC2001 trial in Asian patients with advanced CCA containing *FGFR* alterations, the ORR was an impressive 50% albeit in only 12 evaluable patients, with median progression-free survival of 5.59 months (95% CI 1.87–13.67) [47]. Of the 10 patients that had *FGFR2* alterations specifically, the ORR was 60% with 100% disease control rate. The most common side effects were hyperphosphatemia, dry mouth, stomatitis, and dry skin. 64% of patients had grade 3 or higher adverse events.

## 5. Discussion and Future Considerations

FGFR inhibitors represent a therapeutic breakthrough in molecular subsets of advanced CCA in which treatment options are limited, particularly in the second line and higher settings. As multiple FGFR inhibitors are rapidly under development and increasing routine clinical implementation of these promising class of agents are expected, there remains several key issues that need to be considered prior to optimizing their anticancer potential in advanced CCA.

### 5.1. Side Effects of FGFR Inhibitors

The most common AE in FGFR inhibitor trials in CCA was hyperphosphatemia, which was reported in 60–78% of patients but was rarely grade 4 or 5 [35,36,39,42,44,48]. Hyperphosphatemia is a known on-target side effect of FGFR inhibitors resulting from blockade of FGF23-FGFR signaling in the kidneys and a subsequent increase in renal phosphate reabsorption [50]. In addition to dose reduction, hyperphosphatemia can be managed by initiation of phosphate lowering therapy such as sevelamer when serum phosphate level is greater than 7 mg/dL and by routine phosphate monitoring [51]. Other common side effects included fatigue, stomatitis, dry mouth, diarrhea, eye toxicity, and nail changes, which were also commonly observed in FGFR inhibitor trials in urothelial carcinoma [52]. Within eye toxicities, it should be noted that central serous retinopathy or retinal pigment epithelium detachment is a well-documented FGFR inhibitor side effect. While there are no consensus guidelines, complete ophthalmologic exams prior to therapy initiation and every 1–3 months while on therapy are generally recommended [51,52]. More commonly, patients will experience milder ocular toxicities such as dry eyes that can be managed with as needed or prophylactic ocular lubricants.

### 5.2. Molecular Scope of FGFR Inhibitors in CCA

An important step towards advancing the clinical use of FGFR inhibitors will be determining the molecular scope of their utility. FGFR inhibitors appear to work best in those with *FGFR2* fusions, but less so for *FGFR2* mutations/amplifications or alterations to other FGFR family members [34,38,39,40]. For example, in patients with non-*FGFR2* fusion *FGF/FGFR* alterations, no objective responses have been reported for pemigatinib or infigratinib, though a small pooled cohort analysis may suggest some promise for derazantinib [35,37,50]. As previously mentioned, less than 15% of iCCA cases contain *FGFR2* fusions, thus potentially limiting the scope of FGFR inhibitors. Hopefully, the rapidly expanding repository of genomic data in CCA should pave the way for a better delineation of which *FGFR* mutations sensitize tumors to FGFR inhibitors. Moreover, there may exist CCA subtypes that achieve FGFR pathway overactivation via transcriptional mechanisms and thus could be susceptible to FGFR inhibition even in the absence of *FGF/FGFR* genomic alterations. This hypothesis should be further explored through RNA expression profiling.

### 5.3. Molecular Profiling to Identify Appropriate Candidates

Several methods already exist for detection of *FGFR2* fusions in CCA, each with tradeoffs in cost, sensitivity, and ability to detect novel fusions [53]. While immunohistochemistry (IHC) is readily available and cost-efficient, it can only detect FGFR2 overexpression but not the presence of fusions, and fusion proteins themselves may affect strength of staining and lead to unreliable results. A diagnostic advantage utilized by conventional tests is knowledge that the location of the breakpoint in the *FGFR2* gene appears to be nearly always within intron 17 or exon 18 [53]. Polymerase chain reaction (PCR) is a highly sensitive technique but is limited by the requirement to design primers based on known *FGFR2* fusions, and thus cannot detect novel fusion partners. Break-apart fluorescence *in situ* hybridization (FISH) can detect any *FGFR2* fusion but does not provide a means to identify the fusion partner itself.

More recently, next generation sequencing (NGS)-based techniques have allowed for high throughput and more unbiased detection of genetic alterations in cancer. Using tumor DNA or cDNA as an input, short DNA fragments are generated either by multiplex PCR (amplicon sequencing) or by DNA fragmentation and isolation of targets of interest using biotinylated oligonucleotide probes (hybrid capture sequencing). This is followed by pooled, multiplex sequencing. In particular, hybrid capture-based NGS allows for both the detection of novel *FGFR2* fusions and the identification of the fusion partner. One such test, the FoundationOne^®^ CDX (Foundation Medicine, Inc., Cambridge, MA, USA), was recently FDA-approved on April 17, 2020 as a companion diagnostic for determining pemigatinib eligibility in unresectable CCA in the second-line setting [54]. The assay is able to detect gene rearrangements in *FGFR1*, *2*, and *3*, with hybrid capture of select intronic regions (*FGFR1* intron 1, 5, 17; *FGFR2* intron 1, 17; FGFR3 intron 17) to enhance sensitivity [55]. However, several other commercially available NGS platforms have employed both DNA and RNA sequencing for evaluation of *FGFR* gene rearrangements and fusions. DNA-based methods are more stable than RNA, but detection of novel fusions are limited when compared to RNA-based methods, particularly when large intronic regions are involved [56]. Often, RNA fusion analysis identifies more alterations than DNA fusion analysis, whereas DNA sequencing did not detect any fusions RNA sequencing was unable to [57]. For example, Tempus offers a targeted NGS assay that detects DNA gene rearrangements in *FGFR2* (including the 5′UTR and introns 1 through 17) and *FGFR3*, as well as comprehensive fusion detection by RNA-seq on FFPE tumor tissue. Across 23 patient samples and 4 reference standards, the DNA-seq translocation detection sensitivity was 96.5% (28/29 gene rearrangements within 27 samples). Including RNA-seq, the overall sensitivity of translocation detection increased to 99.9% (29/29) [58]. Ultimately, these targeted NGS assays offer lower costs and higher sequencing coverage compared to unbiased assays such as whole-genome sequencing (WGS), whole-exome sequencing (WES), and whole-transcriptome sequencing (WTS). However, WGS has the advantage of identifying a large number of rearrangements and characterizing breakpoints, including those in non-coding regions, and it is particularly useful for the discovery of novel fusions. WES offers the ability to evaluate DNA contained in all exonic regions of the genome [56]. WTS via RNA next-generation sequencing has the ability to detect rare or novel fusion events better than targeted RNA sequencing or DNA-based methods; one such test is the Caris MI Transcriptome™, which has received FDA Breakthrough Device designation for detection of novel FGFR biomarkers from FFPE tumor tissue [59]. It is worthwhile to note that although more expansive and comprehensive, WGS, WES, and WTS are expensive, require extensive bioinformatics and personnel, and are not currently tailored for routine clinical testing.

### 5.4. Primary and Secondary Resistance to FGFR Inhibitors

Despite the promise of tyrosine kinase inhibitors (TKIs), many tumors ultimately develop mechanisms of resistance upon sustained drug exposure. This phenomenon has been well documented with epidermal growth factor receptor (EGFR) inhibitors in the context of non-small cell lung cancer, in which secondary EGFR mutations can disrupt inhibitor binding [60]. One of the limitations of the FGFR inhibitors currently in clinical trials is that most are ATP-competitive TKIs and are thus susceptible to similar resistance mutations that affect the ATP binding pocket of FGFR proteins. Such mechanisms of resistance to FGFR inhibitors in *FGFR2* fusion-positive CCA were reported in a phase II trial of BGJ398 (Infigratinib) [61]. Pretreatment and post-progression cell-free DNA (cfDNA) was collected from 3 patients who were treated with BGJ398 and experienced initial tumor regression followed by subsequent disease progression. All three patients were found to have between 1 and 5 new *FGFR2* point mutations in their relapse samples that were not present at the pretreatment stage. Strikingly, all three relapse samples harbored a p.V564F gatekeeper mutation, located at the entry of the ATP-binding pocket where FGFR inhibitors competitively bind. The five other amino acid residues for which new mutations were identified were all predicted to be important in stabilizing the inactive conformation of the kinase, to which BGJ398 preferably binds. Thus, mutations to these residues are likely to destabilize the inactive conformation and prevent BGJ398 binding. Similar mutations have been observed in response to pemigatinib within the FIGHT-202 trial as well [31].

Moreover, secondary resistance mechanisms to FGFR inhibitors have been identified. Interestingly, postmortem biopsies from one of the above BGJ398-treated patients demonstrated loss-of-function alterations in *PTEN*, a suppressor of the PI3K/AKT pathway [61]. PTEN loss would result in PI3K/AKT pathway activation despite inhibition of upstream FGFR2 signaling. Another interesting observation, discovered within an interim analysis of the FIGHT-202 trial with pemigatinib, is that 0 out of 5 patients with *TP53* mutations achieved an objective response, compared to 45.2% in the rest of the cohort [62]. However, it should be noted that this observation may result from the overall poor prognosis of *TP53* mutations, as opposed to *TP53* mutations having specific effects on downstream FGFR2 targets.

Fortunately, efforts are already underway to develop next-generation FGFR inhibitors that can overcome primary resistance. Within a phase I study, TAS-120, an irreversible pan-FGFR inhibitor that covalently binds within the ATP pocket, was given to four *FGFR2* fusion-positive patients who had previously been treated with BGJ98 or Debio 1347 [25]. All four patients demonstrated clinical benefit (2 stable disease, 2 partial responses). The authors further confirmed the ability of TAS-120 to overcome resistance in vitro by generating iCCA *FGFR*-*PHGDH* fusion lines with engineered *FGFR2* mutations that had been detected in these four patients after progression on BGJ398 or Debio 1347. TAS-120 maintained a less than 7-fold increase in IC_50_ in all mutants with the exception of the 565F gatekeeper mutation. To address this particular mechanism of resistance, the novel FGFR inhibitor LY2874455 has been developed to overcome gatekeeper mutations [63]. While this provides a roadmap for overcoming resistance mechanisms, unfortunately this particular molecule’s clinical development has been discontinued [53].

As an increasing number of CCA patients are treated with FGFR inhibitors, accurate tracking and detailed characterization of these aforementioned FGFR resistance mutations will be crucial for the continued optimization of FGFR therapeutics. Cell-free DNA sequencing from blood samples represent a non-invasive assay to aid in this endeavor and allow for serial assessment of tumor composition [64].

### 5.5. Biomarkers to Guide FGFR-Directed Therapies

Biomarkers that can quantify the pharmacodynamics of FGFR inhibition would be clinically useful tools to ensure maximal drug efficacy. In the context of urothelial carcinoma, one particular measure that has proven useful in assessing the degree of FGFR inhibition is serum phosphate level as hyperphosphatemia is an on-target side effect. In a Phase II study of erdafitinib in unresectable or metastatic urothelial carcinoma, participants were initially dosed at 8 mg daily, then subsequently increased to 9 mg daily if their serum phosphate level was less than 5.5 mg/dL 14 days after initiation of therapy [50]. Phosphate levels above this threshold correlated with improved response rates in the corresponding Phase I study. Preliminary evidence suggests that this metric can be used in cholangiocarcinoma as well. In the FIGHT-202 trial with pemigatinib, a bell-shaped association between change in serum phosphate concentration and objective response rate was observed, suggesting the existence of an optimal window of phosphate levels to which pemigatinib can be titrated [34]. Future prospective or post hoc analyses of other FGFR inhibitor trials in CCA should be directed towards establishing a clear serum phosphate level cutoff associated with clinical benefit.

### 5.6. Other Avenues to Exploit Therapeutic Potential

Given the promising evidence of FGFR inhibitor efficacy in early CCA trials, the question naturally arises of how FGFR inhibitors can be rationally combined with other systemic therapies to maximize antitumor activity. Interestingly, early preclinical studies have explored the combination of FGFR2 inhibition and anti-PD1 therapy in a lung cancer mouse model harboring a mutant, constitutively active FGFR2 [65]. Dual treatment with erdafitinib and anti-PD-1 resulted in a statistically significant increase in overall survival compared to the control and erdafitinib monotherapy groups. T cell receptor (TCR) sequencing studies revealed that erdafitinib monotherapy resulted in decreased T cell clonality, while combination therapy induced a higher T cell fraction and higher T cell clonality relative to erdafitinib alone. The authors suggest that this finding could be consistent with a mechanism whereby erdafitinib-mediated tumor cell killing facilitates release of tumor cell antigens that expand the T cell repertoire, while addition of anti-PD-1 results in increased T cell infiltration and a focused, more clonal T cell repertoire directed against tumor-specific antigens. These results provide rationale for further exploring combination FGFR inhibition and anti-PD-1/PD-L1 therapy. Moreover, studies in HCC suggest that FGF and VEGF signaling may even upregulate PD-1 expression directly [66]. Future studies will need to be directed towards elucidating this potential synergistic effect.

In another study in urothelial carcinoma cell lines, the FGFR inhibitor derazantinib was found to potently inhibit Colony-Stimulating Factor 1 Receptor (CSF1R) [67]. Inhibition of CSF1R is thought to shift macrophages from the tumor-promoting M2 phenotype to a pro-inflammatory, tumoricidal M1 phenotype that facilitates CD8^+^ T cell infiltration [68]. Based on this immune effect, clinical trials combining CSF1R inhibitors and checkpoint inhibitors are already underway. This study provides further rationale for the combination of FGFR inhibitors and PD-1/PD-L1 blockade, as FGFR inhibitors may therapeutically synergize with PD-1/PD-L1 blockade through “off-target” CSF1R inhibition.

### 5.7. Testing FGFR Inhibitors in the First-Line Setting

With demonstrated safety and efficacy in the second-line setting in CCA, FGFR inhibitors are now poised for testing in the first-line setting. Phase III trials are currently ongoing that are comparing first-line gemcitabine/cisplatin versus pemigatinib (FIGHT-302) [69], infigratinib (PROOF trial) [70], or TAS-120 (FOENIX-CCA3, limited to iCCA only) [71] in patients with unresectable or metastatic CCA and a documented *FGFR2* rearrangement. All three will examine progression-free survival as their primary outcome. While these are exciting and important developments for FGFR inhibitors, there are several caveats that accompany these trials. First, the multitude of trials combined with the relatively low incidence of CCA compared to other cancers may slow the accrual process for each respective trial. Another important consideration in these trials is that *FGFR2* rearrangements must be documented prior to enrollment, and NGS sequencing assays to determine *FGFR2* status can take up to several weeks to result. Thus, it is of the utmost importance to develop assays with faster turnaround times to ascertain *FGFR2* status, especially for patients who cannot afford to wait several weeks for treatment initiation. Otherwise, these trials will likely select for patients who are clinically stable enough to wait for several weeks until treatment initiation, while patients with highly advanced disease will have to pursue gemcitabine/cisplatin in a non-trial setting. Lastly, it is not known whether there is an optimal sequence of administration of gemcitabine/cisplatin and an FGFR inhibitor. Will there be significant differences in outcomes between patients who progress on a first-line FGFR inhibitor then receive second-line gemcitabine/cisplatin and vice versa? These will be important considerations as Phase III trials are ongoing.

## 6. Conclusions

Recent genomic profiling efforts have shed key insights into the molecular landscape of cholangiocarcinoma. The characterization of an FGFR-driven subtype of CCA, most commonly caused by *FGFR2* translocations resulting in constitutive downstream activation, has resulted in the emergence of FGFR inhibitors as a viable treatment option in advanced and metastatic CCA. Promising phase I and II studies have shown clinical benefit in the second-line setting and beyond, with approximately one-third of patients demonstrating an objective response in many of these trials. Moreover, the adverse effect profile is generally quite tolerable, with hyperphosphatemia as the most common side effect. Phase III studies comparing FGFR inhibitors versus gemcitabine and cisplatin in the first-line setting are currently underway. As the use of FGFR inhibitors in CCA increases, several challenges lie ahead. Key issues that warrant further investigation include determining molecular eligibility criteria (which types of genetic alterations in which *FGFR* genes predict clinical benefit), overcoming primary and secondary resistance, and exploring the rational combination of FGFR inhibitors with other systemic treatments such as immunotherapy. Continued investigation of these avenues has the potential to turn FGFR inhibitors into a mainstay of advanced CCA treatment.

## Figures and Tables

**Table 1:** Notable published clinical trials of FGFR inhibitors in advanced cholangiocarcinoma.

Trial, Setting, References	n, FGFR Alterations	Primary Endpoint	Findings
Pemigatinib (ATP-competitive mechanism, selective for FGFR1-3)			
FIGHT-202 (NCT02924376, Phase II), CCA, ≥1 previous systemic therapy [34]	n = 107 *FGFR2* fusions or rearrangements, n = 20 other *FGF*/*FGFR* alterations, n = 18 with no *FGF*/*FGFR* alterations, n = 1 undetermined status	ORR	*FGFR2* fusions/rearrangements: ORR 35.5% (95% CI 26.5–45.4) including 3 CRs, 35 PRs; mPFS 6.9 months (95% CI 6.2–9.6) Other *FGFR/FGF* alterations: 0% ORR, mPFS 2.1 months (95% CI 1.2–4.9) No *FGF/FGFR* alterations: 0% ORR, mPFS 1.7 months (95% CI 1.3–1.8)
Infigratinib [BGJ398] (ATP-competitive mechanism, selective for FGFR1-3)			
NCT02150967 (Phase II), CCA, ≥1 previous systemic therapy, *FGFR2* fusions or rearrangements [35,36,37]	n = 83 *FGFR2* fusions, n = 25 *FGFR* rearrangements	ORR	ORR 23.1% (95% CI 15.6–32.2); mPFS 7.3 months (95% CI 5.6–7.6); mDOR 5.0 months (range 0.9–19.1)
Derazantinib (ATP-competitive mechanism, selective for FGFR1-3)			
ARQ 087-101 (NCT01752920, Phase I/II), iCCA, ≥1 previous systemic therapy or ineligible for first-line chemotherapy [38,39]	n = 29 *FGFR2* fusions; n = 6 *FGFR2* mutations/amplifications; n = 9 no *FGFR2* alterations	Safety and tolerability	*FGFR2* fusions: ORR 20.7% (0 CRs, 6 PRs); mPFS 5.7 months (95% CI 4.0–9.2) *FGFR2* mutations/amplifications: 0% ORR, mPFS 6.7 (95% CI 1.0–14.7) No *FGFR2* alterations: 0% ORR, mPFS 1.5 (95% CI 0.7–N/A)
Debio 1347 (ATP-competitive mechanism, selective for FGFR1-3)			
NCT1948297 (Phase I), advanced solid malignancies harboring activating *FGFR* alterations. Included 9 iCCA patients (1–3 prior lines of systemic therapy) [40,41]	n = 5 *FGFR2* translocations, n = 1 *FGFR1* translocation, n = 1 *FGFR2* mutation, n = 1 *FGFR2* activating deletion, n = 1 *FGFR3* mutation	Safety and tolerability	2/9 PR, 4/9 stable disease
Futibatinib [TAS-120] (covalent irreversible mechanism, selective for FGFR1-4)			
FOENIX-101 (NCT02052778, Phase I), 45 CCA patients (41 iCCA), ≥1 prior systemic therapies (13 received prior reversible FGFR inhibitors) [42]	n = 28 *FGFR2* gene fusions, n = 17 other *FGF/FGFR* alterations	Safety and tolerability	ORR 25% (7/28) in the *FGFR2* gene fusion patients and 17.6% (3/17) in those with other alterations; 30.8% ORR (4/13) in patients who had previously been treated with other FGFR inhibitors; Overall DCR 79%
FOENIX-CCA2 (NCT02052778, Phase II), iCCA patients with *FGFR2* fusions/other rearrangements, ≥1 prior systemic therapy, no prior FGFR inhibitor, ECOG PS 0/1 [43,44]	n = 55 *FGFR2* fusions; n = 12 other *FGFR2* rearrangements	ORR	ORR 37.3%; mPFS 7.2 months; mDOR 8.3 months; DCR 82.1%
Erdafitinib (ATP-competitive mechanism, selective for FGFR1-4)			
NCT01703481 (Phase I), patients with advanced solid tumors for which standard therapy failed [45,46]	Within cohort of 11 CCA patients: n = 3 *FGFR* mutations, n = 8 *FGFR* fusions	Safety and tolerability	CCA patients: ORR 27.3% (95% CI 6–61); mPFS 5.1 months (95% CI 1.6–16.4); mDOR 12.9 months (n = 3)
LUC2001 (NCT02699606, Phase IIa), Asian patients with advanced CCA with *FGFR* alterations [47]	n = 8 *FGFR2* fusions, n = 3 *FGFR2* mutations, n = 1 *FGFR3* fusion, n = 2 *FGFR3* mutations	ORR	Out of 12 response-evaluable patients: ORR 50%; mPFS 5.59 months (95% CI: 1.87–13.67); mDOR 6.83 months (95% CI: 3.65–12.16); DCR 83.3%

FGFR, fibroblast growth factor receptor; FGF, fibroblast growth factor; CCA, cholangiocarcinoma; iCCA, intrahepatic cholangiocarcinoma; ORR, overall response rate; CR, complete response; PR, partial response; mPFS, median progression-free survival; mDOR, median duration of response; DCR, disease control rate; CI, confidence interval; ECOG PS, Eastern Cooperative Oncology Group Performance Status.
